# Reviewing the Significance of Blood–Brain Barrier Disruption in Multiple Sclerosis Pathology and Treatment

**DOI:** 10.3390/ijms22168370

**Published:** 2021-08-04

**Authors:** Rodica Balasa, Laura Barcutean, Oana Mosora, Doina Manu

**Affiliations:** 1 Department of Neurology, University of Medicine, Pharmacy, Sciences and Technology “George Emil Palade”, 540136 Targu Mures, Romania; rodica.balasa@umfst.ro; 2 Neurology 1 Clinic, Emergency Clinical County Hospital Mures, 540136 Targu Mures, Romania; oanamosora.92@yahoo.com; 3 Advanced Research Center Medical and Pharmaceutical, University of Medicine, Pharmacy, Sciences and Technology “George Emil Palade”, 540142 Targu Mures, Romania; doinaramonamanu@gmail.com

**Keywords:** multiple sclerosis, blood-brain barrier, impermeability, disease modifying therapies progression

## Abstract

The disruption of blood–brain barrier (BBB) for multiple sclerosis (MS) pathogenesis has a double effect: early on during the onset of the immune attack and later for the CNS self-sustained ‘inside-out’ demyelination and neurodegeneration processes. This review presents the characteristics of BBB malfunction in MS but mostly highlights current developments regarding the impairment of the neurovascular unit (NVU) and the metabolic and mitochondrial dysfunctions of the BBB’s endothelial cells. The hypoxic hypothesis is largely studied and agreed upon recently in the pathologic processes in MS. Hypoxia in MS might be produced per se by the NVU malfunction or secondary to mitochondria dysfunction. We present three different but related terms that denominate the ongoing neurodegenerative process in progressive forms of MS that are indirectly related to BBB disruption: progression independent of relapses, no evidence of disease activity and smoldering demyelination or silent progression. Dimethyl fumarate (DMF), modulators of S1P receptor, cladribine and laquinimode are DMTs that are able to cross the BBB and exhibit beneficial direct effects in the CNS with very different mechanisms of action, providing hope that a combined therapy might be effective in treating MS. Detailed mechanisms of action of these DMTs are described and also illustrated in dedicated images. With increasing knowledge about the involvement of BBB in MS pathology, BBB might become a therapeutic target in MS not only to make it impenetrable against activated immune cells but also to allow molecules that have a neuroprotective effect in reaching the cell target inside the CNS.

## 1. Introduction

Multiple sclerosis (MS) remains the most frequent cause of nontraumatic disabling disease in young adults [1]. In the last decade, several studies have demonstrated the complex role of the blood–brain barrier (BBB) in immune surveillance of the central nervous system (CNS), the immune preservation of the brain being subjected to the integrity of the BBB. In this light, MS pathogenetic theories have developed. However, MS onset is associated with immune activation in the periphery, followed by immune aggression of the CNS. The onset of the immune-mediated process favours the ‘outside-in’ theory (from the periphery towards the CNS) followed by a CNS self-sustained ‘inside-out’ mechanism (centrally activated lymphocytes against self-myelin return in the peripheral compartment to recruit more proinflammatory lymphocytes) [2,3]. The progressive disability described in most MS patients is related–although not exclusively–to relapses. It may also be secondary to neurodegenerative processes. Both mechanisms are partially related to BBB disruption [4]. 

With MS, the breakdown of the BBB is an early essential step in the initiation and, to a lesser extent, the maintenance of an autoimmune attack against the CNS, which causes demyelination and axonal loss, consequently leading to neurodegeneration and irreversible neurological impairment. The hypothesis agreed by most MS specialists over the last decade proposes that an early focal inflammation secondary to BBB disruption may trigger other pathological modifications, such as neurodegeneration and diffuse inflammation, to occur [5]. Breakdown of the BBB is characterised by peripheral activation and transendothelial migration of the T lymphocytes followed by sudden clinical worsening if the BBB is penetrated in an eloquent neurologic area [6]. Magnetic resonance imaging (MRI) highlights active demyelinating intracerebral plaques in vivo, as a consequence of BBB disruption, with the use of gadolinium (Gd) [7,8]. 

This review discusses the significance of BBB disruption, with a special focus on the mechanism of action (MoA) of the new molecules that bypass the BBB. In addition to the peripheral decreased effect of Th and B lymphocytes, which contribute to BBB disruption by crossing it, the new molecules have a central effect, with particular implications for MS.

Increased knowledge of the BBB involvement in MS pathology, as well as of disease modifying therapies (DMTs’) effects after crossing the BBB, may result in improved drug selection for the clinical evolution in treating MS patients by using better therapy for BBB malfunction and uncontrolled neurodegeneration.

## 2. The Correlation between the Structure and Function of the BBB in Both Normal and Demyelinating Brains

The BBB is a complex, highly specialised structure of brain endothelial cells. Cere-bral endothelial cells, together with pericytes and their adjacent basal lamina that support, surround and connect with astrocytes and perivascular macrophages, constitute the neurovascular unit (NVU) [9]. Endothelial cells are very closely connected by tight junctions (TJs) and adherent junctions (AJs). TJs are composed of transmembrane proteins, such as occludin and claudin-5, and cytoplasmic proteins, such as ZO-1, -2 and -3, as well as being supported by an actin cytoskeleton. AJs are composed of VE-cadherin and catenins [10,11]. 

The BBB serves as a ‘physical barrier’ to blood cells and other molecules. It also maintains brain homeostasis by controlling nutrient, water and molecule exchanges and purging waste products from the CNS [9]. Under normal conditions, the BBB endothelium, unlike the rest of the capillary endothelium, lacks transendothelial fenestrations but it has a larger transport system for better control of the transcellular flux [12]. The transcellular transport (characterised by rare transcytosis vesicles, very low pinocytotic activity and an active membrane ATP efflux for lipophilic substances) and the para-cellular transport (for hydrophilic molecules) provide for the passage of cells and various other substances through the BBB; they are strictly controlled and take place with regulated energy consumption [9,10,11,12,13].

**Figure 1:**
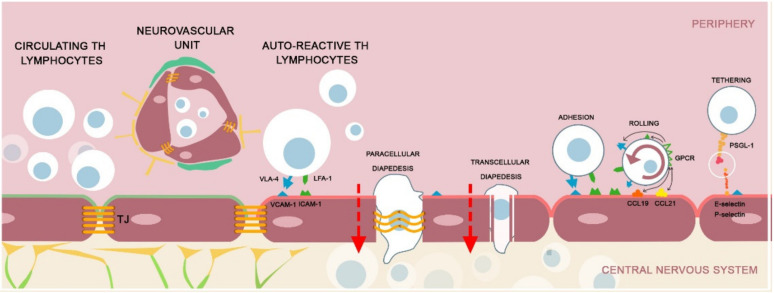
A brief schematic presentation of the pathophysiology of MS regarding the disruption of the BBB. The NVU is composed of endothelial cells, astrocyte end-feet and pericytes [9]. The endothelial cells are interconnected by TJs [10,11]. In healthy individuals, the integrity of the BBB maintains the immune circulating cells in the peripheral blood compartment. In MS, the auto-reactive Th cells will extravasate across the vascular endothelium in a complex manner. **Tethering:** The peripheral lymphocytes express P-selectin glycoprotein ligand-1 (PSGL-1) that interact with the ligand molecules expressed on the endothelial cells (E and P-selectins). **Rolling:** The contact between the lymphocyte and the endothelium is preceded by the rolling beside the vessel wall–a transient, reversible process. The endothelial cells express various chemokines (CCL21, CCL19) that will activate the G protein coupled receptor (GPCR) on the surface of the lymphocyte and stimulate the expression of integrins, very late antigen-4 (VLA-4) and lymphocyte function associated antigen 1 (LFA-1) [21,22,23,24]. **Adhesion:** The lymphocyte will adhere to the endothelial cells by coupling the surface adhesion molecules (VLA-4 and LFA-1) with the endothelial cell receptors (VCAM-1 and ICAM-1). After coupling, the lymphocytes will transverse the BBB by **transcellular or paracellular pathways** [25]. The activated T lymphocytes have the capacity to alter the inflamed BBB and create a transendothelial pore by modelling the caveolin-1 and transverse by a transcellular pathway. In the paracellular breakthrough, the T lymphocytes remodel the TJs by altering the connective molecules (occludin, claudin) and create a permissive ‘fenestration’ [26,27,28,29].

In MS patients, the T lymphocytes are activated in the peripheral compartment, and, as an essential step, they infiltrate the CNS, secondarily triggering a central immune response that can further auto-maintain itself, leading to myelin and axonal damage [14]. After more than a century of microscopic studies and clinical research, the etiology of MS remains largely unknown. The latest findings in treating MS with DMT or with immune reconstruction therapy showed that BBB disruption and lymphocyte trafficking are among the most important pathological processes [15]. The mechanisms involved are not limited to acute demyelinating brain lesions: in ‘inactive’ regions, as well as in regions with normal-appearing white matter, histopathological studies have demonstrated BBB abnormalities [6,16]. Leukocyte-derived proinflammatory cytokines activate the endothelial cells in the BBB and induce the expression of additional adhesion molecules, leading to a self-sustained CNS infiltration of more immune cells [17]. The process of lymphocyte ‘walking-through’ the endothelium by the lymphocytes is facilitated by their interaction with chemotactic factors and cell adhesion receptors. Activated lymphocytes, mainly T helper (Th) cells, slow their flow speed due to the interaction of their adhesion receptors from their surface (integrins such as very late antigen 4 or α4-integrin, leukocyte functional antigen) with adhesion molecules (selectins) from the surface of the inflamed endothelium [18]. In addition, drastic morphological changes of the lymphocytes allow tethering and rolling at the level of the junctional proteins, leading to the onset of transendothelial selectin-mediated migration. This multi-step process is orchestrated by numerous molecules, such as vascular cell adhesion molecules (VCAM), intracellular adhesion molecules (ICAM), cytokines and inflammatory mediators, together with endothelial alteration of transcellular and paracellular transport (Figure 1) [19,20].

Structural changes in the endothelium of the BBB further contribute to a sustained inflammatory response. Numerous factors modify the BBB endothelium in MS patients. For example, the interaction of monocytes with the brain endothelium produces reactive oxygen species (ROS) that subsequently alter the impermeability of TJs. The integrity of the BBB is further compromised by the extravasation of Th cells into the CNS [30]. After protrusion, the transmigration of leukocytes is facilitated by chemokines. The action of matrix metalloproteinases (MMP) facilitates lymphocyte passage through the glia limitans towards the endoneural space. Once settled, the Th cells interact with different auto-antigens in the CNS and a rapid clonal expansion follows. Ultimately, the immune response is amplified and self-sustained, triggering a cascade of inflammatory, demyelinating and neurodegenerative events. Cytokines, chemokines, proteases, nitric oxide, free radicals and various toxic mediators are released into the central compartment with a culminating effect on axonal damage. The resident CNS cells, such as glial cells, are activated toward a proinflammatory state, and this initiates antigen presentation to Th cells [6].

The BBB is a complex barrier, representing a conceptual multilayered structure rather than a simple fence. Cerebral blood capillaries have a different structure from the rest of the body’s capillaries. For a larger occlusion, the brain endothelial cells are sealed with TJs that lack pores. In the CNS and at the level of the blood cerebrospinal fluid barrier (BCB) level, epithelial cells of choroid plexus are connected to each other by TJs and are attached to the blood vessels via basement membrane proteins. On the blood-facing side, these epithelial cells are permeable for molecules from the bloodstream, as endothelial cells in choroid plexus lack TJs and allow molecule diffusion through the fenestrated capillaries. The choroid plexus is part of the circumventricular organs, a structure lining the 3rd and the 4th ventricle [31]. This particular structure allows for the first demyelinating wave of Th lymphocytes (mainly Th17 cells) that enters the CNS due to the CCR6 receptor interaction with the CCL20 ligand present on these epithelial cells. This first wave facilitates the CNS entry for the second demyelinating wave, independent of the CCR6 receptor, and contributes to the BBB breakdown [30,32,33]. Schulz M, et al. reported in murine EAE studies that a significant number of autoreactive Th lymphocytes are found at the level of the choroid plexus, together with up-regulation of major histocompatibility complex I and II, adhesion molecules and microglial activation. Apart from the choroid plexus epithelium, BCB comprises an endothelium with less permeability and represents the transition towards the endothelium found in the BBB [34]. As the surface of the BBB is significantly larger than that of the BCB, the rest of the review will focus on the dysfunctionality of the BBB in pathological conditions found in MS.

The molecular flux across the BBB involves several transport mechanisms. A pas-sive mechanism allows for the passage of only lipid-soluble molecules. The active transport, driven by ATP-efflux pumps, mainly for xenobiotics and endogenous metabolites, limits the permeability of therapeutic agents. The transcellular carrier-mediated transport allows the passage of vitamins, hormones, amino acids, fatty acids, nucleotides, amines and choline. The receptor-mediated and adsorptive-mediated transcytosis is a bidirectional mechanism for transporting large molecules such as peptides and insulin-receptors. Essential omega-3 fatty acids pass by endothelial facilitator, superfamily-assisted transport [6,35].

In MS pathology, the disruption of the BBB has several characteristics. It is heter-ogeneous and more frequently observed during the first years after disease onset when the clinical manifestations are represented by MS relapses and Gd+ lesions on T1 MRI. Subsequently, depending on the patient, the progression of brain atrophy is related less to BBB breakdown and more to self-sustaining in the central compartment. It is also transient, and recurrence may be observed in different locations, but may also occur in the same spot (evident on MRI as lesions with a ring enhancement or with a blurred rim) [36]. BBB breakdown, reflecting the degree of MS aggressiveness at the onset of the disease, is a criterion to classify the disease as active and to select the appropriate therapy. The first stage of MS, as was initially outlined by Steinman in 2001 and later by Leray in 2010, is characterised by BBB disruption and is more receptive to therapy. This two-stage disease concept (the first stage being dependent on focal inflammation while the second is independent) is supported by MRI and clinical data, and, most importantly, by therapeutic evidence. Early and more effective therapeutic interventions facilitating fewer focal lesions (relapses or new MRI lesions) with an improved or constant neurological state is maintained during short-term follow-up [5,37]. During the neuroinflammatory phase, the BBB structure is modified in certain regions of the endothelium, before becoming irregular and triggering leukocyte extravasation from the bloodstream. The BBB disruption is recurrent at different time intervals and triggered by unknown factors. As described over a century ago, demyelinating lesions in MS have a topographically variable disposition with topographical differences. The majority of demyelinating plaques are situated at the perivenular level, as observed on MRI as the central vein sign. The involvement of BBB disruption in demyelinating cortical plaques due to subpial demyelination has been debated, considering that contrast enhancement is rarely observed at this level [38].

The most important research breakthrough regarding BBB behaviour in MS was achieved using MRI on a large scale. The early disruption of BBB in MS was documented by MRI studies using contrast enhancement. The disruption of the BBB is not limited to contrast Gd+. Previous research has described abnormalities in the permeability of the BBB in normal-appearing white matter in selected MS cases. Recent MRI studies using 3T dynamic contrast-enhancement showed global BBB disruption as an early prognostic factor for the conversion of optic neuritis to MS [2,39].

## 3. Metabolic Changes in BBB Cells

The BBB and the BCB form an integral network with a dual role: to protect the CNS from various damaging substances in the bloodstream and, at the same time, to allow for metabolic exchanges that are vital for CNS homeostasis.

Active transport of substances through the BBB is an energy-intensive task. BBB components, primarily the endothelial cells, are adapted for ideal CNS function. They select and actively transport various molecules using specific transporters. Thereby, these cells have an increased metabolic demand. In 2020, Sheikh et al. described the effect of sera from relapsing-remitting MS (RRMS) patients on endothelial cells of the BBB, showing that energetic and metabolic changes, such as altered oxidative respiration, release of ROS and down-regulation of glycolytic pathway occurred within these cells. Circulating factors in the serum of RRMS have an immune-metabolic impact and determine alterations in the metabolism of BBB endothelial cells (impaired glucose metabolism and impaired in mitochondrial function) [40]. One of the most important alterations linking impaired BBB endothelial cell metabolism with cerebral vascular impairment (see below) in MS is the structural and functional deterioration of the mitochondria. In response to the serum of RRMS patients, the mitochondria become significantly hyperpolarised–a phenomenon that may be an origin of ROS [6,9,40].

Finally, these metabolic pathological processes lead to a perturbed BBB function with impaired transcellular transport of molecules and endothelial junction organisation. The capacity of endothelial cells to provide the best energy supply for ideal BBB function is a significant phenomenon. Any free radical production or mitochondrial dysfunction reverberates on the organisation of junctional molecules (claudin, occluding, cadherin) and enhances BBB permeability due to energetic stress [11,41].

MS, as a chronic metabolic disorder, has been largely debated, with the dysfunction of the metabolism of lipids in the endothelial cells hypothesised. The deficient lipid metabolism appears to manifest during MS relapse and may be triggered by environ-mental factors. The potential result is an increase in oxidative stress with an increased inflammatory response in certain regions of the BBB [42,43].

BBB disruption is not only directly and immediately deleterious in MS pathology by allowing the entrance of proinflammatory cells into the CNS, it may also have delayed secondary effects on MS resulting from the continuous self-maintained proinflammatory and neurodegenerative effects of some cells. As an example, the Th 17 cells, with the high expression of granzyme B, attain increased migratory capacity on arrival in the CNS and continue to stimulate demyelination and extensive axonal degeneration through the following: (1) direct interaction with myelin oligodendrocyte glycoprotein; (2) Th 17 cells secretion of IL-17 and IL-21 and the stimulation of the development of ectopic lymphoid structures; and (3) a decrease in the remyelinating processes by the inhibition of oligodendrocytes [44,45].

## 4. Microvasculature: The Involvement of NVU in MS Pathology

The modern approach to BBB disruption in MS considers the vascular changes at the level of the NVU, which are found early and influenced by the same environmental (pathogens such as Ebstein–Barr virus, smoking, high salt intake, and vitamin D deficiency) and genetic (e. g., APOE and IL7R) risk factors underlying MS pathogenesis. The BBB is considered the central part of the NVU, and its function and cerebral perfusion are closely linked and sometimes superimposable. Spencer et al. highlighted that the NVU changes play a central role in MS pathogenesis – an important initiator of events that contribute to or trigger the development of the immune cascade at the level of the CNS [2].

The NVU is represented by certain cells (endothelial cells of the BBB, neurons, as-trocytes, pericytes and myocytes) and extracellular matrix components. This structure ensures homeostasis, which supplements the blood to the cerebral tissue under certain conditions. This mechanism is called cerebral hyperemia; it is a hemodynamic response that is important in brain homeostasis and supplies the brain with the ideal amount of oxygen and nutrients (glucose). NVU harmoniously couples cerebral blood flow with neural activity in different regions of the brain. When needed, vasodilation and vasoconstriction occur [9,11,12].

Pericytes–cells embedded in the basement membrane of the cerebral microvessels–are uniquely situated in the NVU between the endothelium, neurons, and astrocytes. These cells receive signals from neural cells and generate functional responses (regula-tion of permeability, clearance of toxic metabolites, hemodynamic responses and neu-roinflammation). The inner layer of the NVU is formed by endothelial cells. Under pathological conditions such as MS, increased NVU permeability is secondary to endothelial cell dysfunction. In developing MS lesions, fibrinogen deposition is observed as a marker of endothelial permeability. The increase in the permeability of the NVU is mainly due to changes in the endothelial cell layer, which stimulates the transcellular immune migration (proinflammatory chemokines that upregulate adhesion molecules) and the paracellular route (TJ abnormalities materialised with fibrinogen leak). Perivascular astrogliosis and the retraction of astrocytic end-feet contribute to the exposure of the central compartment to cells and substances from the bloodstream [9,11].

Certain similarities between MS and stroke have been reported. At the cellular level, they both provoke demyelination, axonal injury and neurodegeneration. Neuroinflammation, glial proinflammatory activation and Th-cell influx are common elements of these pathologies. The involvement of NVU in MS raises the importance of cerebral hypoperfusion in MS pathology and could be a possible treatment target. Some DMTs that are used for MS treatment, such as natalizumab or fingolimod, are the subject of research on ischemic stroke patients. Demyelinating MS lesions show the degeneration of oligodendrocytes–cells that are also susceptible to hypoxic injury. Hypoxia in MS may be facilitated by NVU malfunction secondary to mitochondrial dysfunction (inability to properly use oxygen) [46].

Demyelinated axons have a reduced speed of impulse transmission due to the re-distribution of sodium channels as well as mitochondrial changes. Mahad et al. suggested that MS may be a ‘mitocondriopathy’, as mitochondrial dysfunction is not only observed in lesions but also normal-appearing white and grey matter [47]. Other components of the NVU are affected by MS as a consequence of ischemia: pericytes experience contraction and apoptosis. This phenomenon determines capillary constriction that promotes increased BBB damage. Global hypoperfusion in both the white and grey matter was associated with active MS with cognitive dysfunction [48]. Troletti et al. suggested that BBB disruption may be secondary to the endothelial-cell phenotype change by de-differentiation into mesenchymal cells–a process also observed in brain disorders, such as MS [49].

As a possible link between cerebral hypoperfusion in MS and BBB disruption, drugs, such as statins (used in cerebral ischemia), showed some effect in reducing cortical atrophy in progressive MS [50]. The role of statins for MS therapy is controversial. EAE murine studies reported immunomodulatory and neuroprotective effects, such as modulation of the inflammatory responses towards the anti-inflammatory Th2 and decrease of the inflammatory cells’ passage through the BBB. Statins may promote remyelination by augmenting the differentiation of the oligodendrocyte progenitor cells. A meta-analysis performed by Pihl-Jensen et al. investigated the role of statin in MS and revealed no clinical benefit from monotherapy or association therapy with interferon-beta [51,52,53,54]. Encouraging results appeared from a phase 2 clinical trial, MS-STAT, which enrolled secondary progressive MS patients who were treated either with simvastatin or placebo over a period of two years. In the statin-treated group, brain atrophy was 43% lower compared with the placebo-treated group [55]. A currently recruiting trial, MS-STAT phase 3, is aiming to confirm disability progression in patients with progressive MS treated with statins [56].

## 5. BBB in Progressive Forms of MS

The absence of, or significant increase in, Gd+ lesions and the lack of a clinical re-sponse to current DMTs in progressive forms of MS may suggest that the ‘compartmentalised inflammation’ in the CNS is secondary or it follows the integrity of the BBB after a variable period of MS evolution. Unfortunately, this is an overly simplistic approach because fibrinogen deposits were found in the cortex in progressive forms of MS, a consequence of TJ abnormalities. These findings correlate with neuronal degeneration in patients with MS. In addition, TJ abnormalities were observed in tissues, such as normal-appearing grey matter in progressive MS forms, which could indicate BBB disruption [2,57,58,59].

Progressive forms of MS have a spinal predominance of lesions, but, notably, the blood–spinal cord (BSC) barrier represents a more permeable structure than the BBB. This may explain why spinal cord symptoms and lesions are common during the early stages of MS and persist during the progressive phase, with no evidence of BSC barrier disruption. This aspect is less understood [60].

There are three different but related terms that dominate the ongoing neurodegenerative process in progressive forms of MS that are related to BBB disruption. The first is the progression independent of relapses (PIRA), which is independent of MRI activity and was initially used by Kappos et al. when analysing the effect of natalizumab on disability. The authors noted that some treated patients were deteriorating from a neurological standpoint although they no longer had clinical and MRI relapses. In conclusion, PIRA represents a clinical worsening independent of disease activity in the presence of an intact BBB. PIRA contradicts previous theories that considered that disability without inflammation was not ‘true progression’. PIRA is rarely found in relapsing forms of MS, and it is not greatly influenced by current DMTs. PIRA is not influenced by relapses, but its possible relation to the chronic MRI evolution should be considered in the future. The next term that requires a clear definition is the progression independent of MRI activity (PIMA) [61]. The concept of ‘no evidence of disease activity’ (NEDA) evolved over the last decade, from the criterion of no disease activity (stable neurological examination, no relapses, no new or enhancing MRI lesions) as the gold standard for therapy to more complex criteria to affirm that a patient has NEDA. The most recent NEDA 8 adds other signs of disease progression, such as cognitive decline, CSF light chain neurofilament, loss of brain volumes, patient-related outcome, and oligoclonal bands, to the previous criteria [62]. Elliot et al. considered progressive clinical evolution in patients with no MRI Gd+ activity to be secondary to smoldering demyelination or the so-called ‘silent progression’ (after Cree et al.), which is correlated with brain atrophy independent of relapses [63,64].

## 6. Chronic MS DMTs with Central Effect beyond the BBB

The last two decades were marked by remarkable progress in the long-term treatment of MS. The DMTs available today mainly influence the inflammatory phase of the RRMS but differ in their MoAs and potential to penetrate the BBB. The majority of DMTs influence the peripheral immune compartment, suppressing, at some point, the immune attack of proinflammatory leukocytes oriented towards the BBB. In this review, we will focus on the DMTs that have already been approved for the treatment of MS and act on the BBB, but with a special interest in those that, at least theoretically, exert certain beneficial effects directly on the CNS cells. Although they have different MoAs, the recently approved DMTs may have beneficial effects on the progressive form of MS (especially siponimod), where the leading pathological mechanisms underlying progression rely mainly on the central compartment [65].

The effects of DMTs on BBB cells and the central compartment have at times been overlooked. This is not due to negligence but to the difficulty of investigating this aspect in vivo and in vitro. What is interesting and surprising is that some of the DMTs have possible unanticipated effects beyond the BBB. Unfortunately, we can say that MS re-mains incurable, regardless of the treatment. The possibility of a DMT influencing the natural history of MS is an ongoing dilemma.

The DMTs that might exert their direct mechanisms of action in the central compartment are listed below.

Interferon-beta (IFN-β) therapy has been used for RRMS treatment for almost three decades (since 1993). IFN-β was the first DMT approved; its main effect was the immunomodulation of the peripheral proinflammatory cells and the resultant downregulation of the inflammatory cytokines [66,67]. There are two types of IFN-β: IFN-β-1a and IFN-β-1b used in RRMS, with the latter having been approved for secondary progressive MS [68]. The anti-inflammatory effects of IFN-β include a decrease in T-lymphocyte proliferation. IFN-β treatment blocks the in vitro migration of leukocytes through the endothelial cells of the BBB from the peripheral blood by reducing the histamine-induced permeability, stabilising the BBB, and changing the molecular structure of TJs [69]. The integrity of the BBB is dependent on the integrity and placement of TJs. Kuruganti, et al. demonstrated that IFNβ induces morphological changes in the aggressed BBB, by translocating the zona occludens multiprotein junctional complexes in order to reestablish the selective permebility. Also, Kraus, et al. reported on murine studies that BBB TJ integrity was up-regulated secondary to IFNβ administration [69,70,71].

Glatiramer acetate (GAs) constitute a group of synthetic peptides with a structure that resembles the myelin basic protein. The course of MS relies on an increase in the production and activation of anti-inflammatory Th2 cells and related cytokines. GA suppresses the activation of myelin-reactive Th1 cells through the downregulation of antigen-presenting cells that express major histocompatibility complex (MHC) molecules on their surface. GA Th2 cells cross the BBB and modify the function of the CNS by producing a bystander suppression of the Th1 cells that are myelin-reactive and secondarily lowering the secretion of proinflammatory cytokines. In addition, GA Th2 cells, on arriving in the CNS, achieve a neuroprotective effect by secreting neurotrophic factors [72].

Natalizumab is a monoclonal antibody specially designed to prevent the entry of lymphocytes into the BBB. This is achieved by blocking α4-integrin from the surface of lymphocytes, making it impossible for them to bind to the VCAM from the surface of the BBB’s endothelial cells. It is widely used for treating RRMS [9,73]. With natalizumab, lymphocytes cannot adhere to the BBB. Unfortunately, this substance, by inhibiting any immune surveillance against viral leukoencephalopathy produced by the John Cunningham virus, may provoke a potentially deadly complication (progressive multifocal leukoencephalopathy) [74]. Numerous published studies have shown multiple peripheral effects of natalizumab, but no direct effects in the CNS [75,76]. Natalizumab therapy indication was not extended for secondary-progressive MS. The results of the phase 3 ASCEND study showed no difference in disability progression. The primary and secondary endpoints of the study were not met [77].

The combination of peripherally acting monoclonal antibodies and BBB-crossing molecules (as described below), is promising, at least in theory, for better control of MS pathological processes and a cessation of disease progression. Compartmentalized CNS inflammation, with the discovery of lymphoid-follicle-like structures contributing to the irreversible disability in MS, makes the success of treatment difficult to imagine without the use of active molecules in the brains of our patients [78].

Dimethyl fumarate (DMF) is an oral DMT that was approved in 2013 as a first-line therapy in RRMS patients. Numerous trials have shown that DMF reduces clinical MS activity. The main immunomodulatory effect of DMF is on the peripheral immune system and can be divided into two main pathways regarding the influence on the nuclear derived 2-related factor (Nrf2, an important transcription factor that maintains intracellular redox homeostasis). The effects of DMF dependent on the Nrf2 pathway in the periphery are as follows: an increase in the expression of antioxidants, detoxifying enzyme genes, regulatory T cells, natural killer cells and plasmacytoid dendritic cells, and a decrease in CD8+ T cells, B cells and type 1 myeloid dendritic cells [79,80]. The effects independent of the Nrf2 pathway rely on the activation of the hydroxyl carboxylic acid receptor [2]. DMF reduces neutrophil infiltration in the CNS as well as reduces inflammatory cytokines and the function of some antigen-presenting cells. It also increases the levels of cAMP that can diminish T-cell proliferation, promote the secretion of anti-inflammatory cytokines such as IL-10, and decrease the secretion of proinflammatory cytokines (TNF-α, IL-2) [81,82].

**Figure 2:**
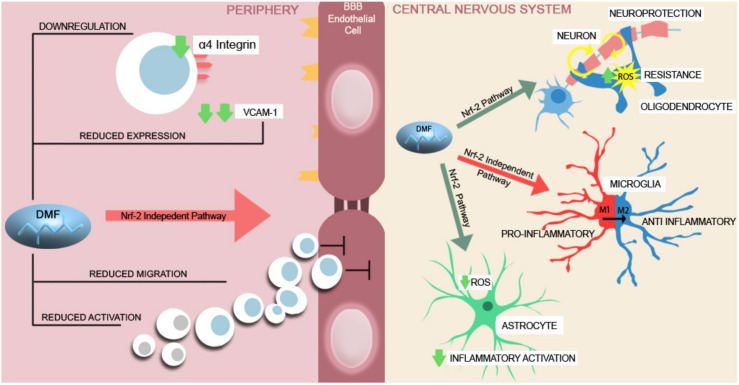
A brief schematic representation of DMF’s mechanism of action. In the peripheral compartment, DMF induces changes in the immune response by decreasing the activation and migration of Th lymphocytes. It further alters the transendothelial migration across the BBB, reduces the expression of the adhesion molecules, such as VCAM-1, and downregulates the α4 integrin on the lymphocyte surface [81,82,84,85,88,89]. Inside the CNS, DMF carries Nrf-2 dependent neuroprotective effects upon the neurons and oligodendrocytes by increasing the ROS resistance of the neurons and glial cells and upon the astrocytes by decreasing the proinflammatory cytokine secretion and intracellular ROS production [86,90,91]. In proinflammatory activated microglia, DMF reduces the production of proinflammatory mediators and stimulates the shift from a M1 proinflammatory state to a M2, anti-inflammatory state [86,92].

In addition to changing the immune composition towards an anti-inflammatory state and changing the immune cell phenotype (by suppressing differentiation of dendritic, neutrophils, pathogenic Th1 and Th17 cells, and augmentation of natural killer cells), DMF is presumed to have a double effect on the CNS: it reduces immune migration and has direct effects on neurons and glial cells. The effects on immune migration are achieved through a non-Nrf 2 pathway: downregulation of α4-integrin, decrease in the expression of intestinal and BBB adhesion molecules and the suppression of the activation state/migratory activity of lymphocytes. DMF has direct effects on CNS cells, including a neuroprotective effect through an Nrf 2 pathway and the activation of the antioxidant pathway in neurons and neural stem/progenitor cells. DMT affects the metabolism of oligodendrocytes (one of the main centres of degeneration in MS), subsequently preserving the myelin sheath. This protective effect is mediated by the Nrf 2 pathway and consists of protection from oxidative stress by increasing their resistance to ROS [83,84,85]. DMF changes the phenotype of microglia, from an M1 (with important proinflammatory and destructive effects predominating in MS) to an M2 anti-inflammatory type through an independent Nrf pathway. The suppression of activated microglia preserves synapses and has a neuroprotective effect. DMF, by activating the Nrf 2 pathway, can reduce (by decreasing NO synthase) the inflammatory activation of astrocytes (where the strongest Nrf 2 expression is found) (Figure 2) [82,86,87]. 

Laquinimod is an investigational CNS-active immunomodulator. We present its mechanism of action, although it has not yet been approved for MS treatment, as it diffuses freely across the BBB and does not need active transport due to its small dimension that allows for passive crossing. According to the available studies, laquinimod is by far the most potent DMT that influences brain atrophy. Filippi et al. showed that laquinimode significantly reduces brain atrophy and contributes to its neuroprotective role. In the peripheral compartment, laquinimod downregulates the function of myeloid cell populations that have the property of antigen presentation, thus decreasing the proinflammatory T cell responses. It stimulates a Th2 shift on cells with anti-inflammatory effects, increasing the prevalence of T regulatory cells. Laquinimod directly acts on the BBB endothelium by diminishing the expression of adhesion molecules and, consequently, decreasing the permeability of activated leucocytes. Laquinimod promotes the tightness of brain endothelial cells and ameliorates the integrity of the BBB [93,94].

**Figure 3:**
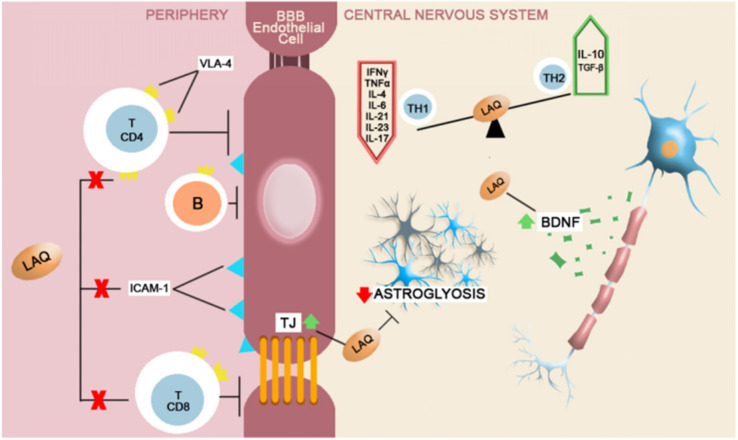
A brief schematic representation of Laquinimod’s mechanism of action. In the peripheral compartment, Laquinimod inhibits the lymphocytic differentiation for CD4+ and CD8+ Th cells, limits the B cells passage and the lymphocytic endothelial adhesion by down-regulating VLA-4 and ICAM-1 [97,101,102,103]. The neuroprotective effects of laquinimod are dependent on BDNF up-modulation with secondary reduced axonal loss. It also reduces astroglyosis and oligodendrocyte apoptosis and reduces the expression of proinflammatory Th1 cytokines, while augmenting the T2 anti-inflammatory response [104,105,106].

Laquinimod in the CNS compartment has the following effects that may be neu-roprotective: it downregulates the astrocytic response with effects on MMP expression and diminishing astrogliosis, stimulates the induction of brain-derived neurotrophic factor (BDNF), decreases microglia activation and prevents synaptic alterations. All these ef-fects contribute to neuronal and glial cell protection from any structural damage that leads to neurodegeneration [95]. According to results from experimental studies, laquinimod acts directly on the NVU (Figure 3) [96]. 

Due to contrasting results in clinical trials, the molecule has yet to receive approval for the treatment of MS. In ALLEGRO, a phase 3 clinical trial, laquinimod was proven to decrease the relapse ratio and disability progression [97]. The most significant results pointed towards reducing brain atrophy, but this effect did not last beyond 12 months [93]. Early EAE studies regarding Laquinimod safety did not reveal cardiac involvement but the follow-up phase 3 CONCERTO (RRMS) and ARPEGGIO (progressive MS) trials led to a discontinuation of the high dose laquinomod due to cardiotoxicity. Their results also failed to show an impact on progression of disability. Even if a significant effect was noted upon reducing the cerebral lesion burden, this was maintained for a short-term only and the finding is not a reliable substitute for assessing neuroprotection [97,98,99,100,101].

**Figure 4:**
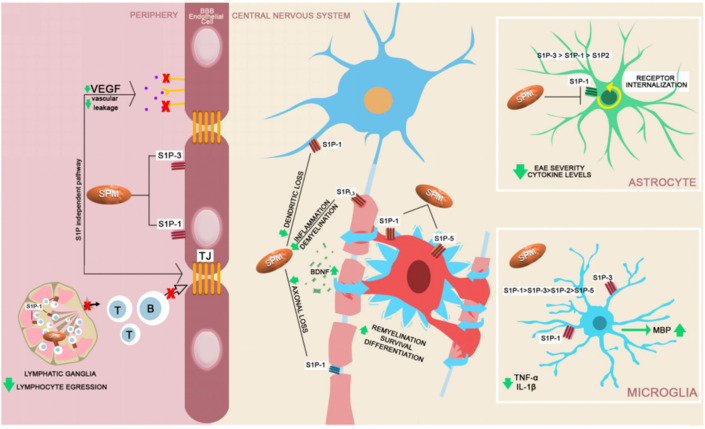
A brief schematic representation of fingolimod’s mechanism of action. In the periphery, fingolimod (sphingosine phosphate receptor modulator–SPM) acts upon the sphingosine 1-phosphate receptors (S1P1, S1P2, S1P3, S1P4, S1P5) [101]. In the lymphatic ganglia, SPM antagonize the S1P1 expression and blocks the lymphocytic egression into the circulation (T and B). SPM blocks the expression of S1P1 and S1P3 on the surface of the endothelial cells, reducing lymphocyte transmigration. Immune cell passage at the level of the BBB is reduced secondary VEGF reduced expression [109,110,111,112]. Inside the CNS the S1P receptors are expressed by the majority of neural cell lineages [113]. S1P1 and S1P5 modulation enhances oligodendrocyte function, survival, differentiation and secondarily boosts the remyelination [114,115]. Neuronal function is directly sustained by modulation of S1P1 and S1P3 receptors and by the up-regulation of BNDF expression, with neuroprotective effects [113,116]. The astrocytes, secondary to S1P1 modulation were proven to decrease EAE severity in murine studies and decrease the levels of proinflammatory cytokines [117,118]. fingolimod and SPM reduce proinflammatory cytokine production from microglia and increase the secretion of myelin basic protein (MBP) after a demyelinating event, promoting remyelination [119,120].

Fingolimod is the first oral DMT approved for MS treatment since 2013. It is the first sphingosine 1-phosphate (S1P) receptor modulator. S1P belongs to the lysophospholipid family and is a natural lipid. Receptors for S1P are found on numerous cells, including lymphatic ganglions, as well as in all cells from the CNS. The S1P-receptor family is composed of five members: S1P1, S1P2, S1P3, S1P4 and S1P5. Neurons, astrocytes, oligodendrocytes and microglia mainly present with two or more S1P receptors, other than S1P4. Fingolimod and all compounds from its class can cross the BBB, and they have an important direct effect on the CNS. In the peripheral compartment, fingolimod inhibits the egress of the autoreactive lymphocytes from the lymph nodes, making it a candidate for other autoimmune or cerebrovascular diseases. Inside the CNS, depending on which cells it acts on, fingolimod has the following effects: on neurons, it reduces dendritic spine loss, restores neuronal function, and protects against excitotoxic death; on oligodendrocytes, it promotes survival and enhances remyelination and differentiation; on astrocytes, it inhibits proinflammatory cytokine production, stimulates cell migration, decreases astrogliosis and reduces ceramide production (reactive astrocytes are the leading cellular source of increased ceramide in MS); on microglia, it reduces microglial activity and enhances microgliosis. In addition, this group of DMTs decreases BBB leakage (induce AJ) and prevents or restores synaptic defects (Figure 4) [107,108,109]. 

Siponimod is a selective S1P receptor modulator (S1PR1 and S1PR5) approved for RRMS, but it is also the only DMT approved for active SPMS. Ozanimod modulates S1PR-1ly primarily, and S1PR-5 to a lesser extent. Compared with the previous two members of the same class, Ozanimod facilitates a quicker lymphocyte reconstitution after discontinuation. Ponesimod is a selective S1PR-1 modulator that has been evaluated by authorities and recently received approval for active RRMS treatment by the EMA [121,122,123].

Fingolimod and natalizumab are considered for ischemic stroke (IS) treatment. Neuroinflammation is common both in MS and IS but has two different origins: thromboinflammation and ischemia in IS and autoimmunity in MS [46,124]. The functional prognostic of IS patients is largely dependent on the NVU involvement. NVU modulates BBB regulation, cell preservation and mediates the inflammatory immune responses and repairment [125]. BBB inflammation secondary to focal cerebral ischemia facilitate proinflammatory Th lymphocyte aggregation and transmigration, which in turn maintains local inflammation [126]. Fingolimod, acting against S1P1 receptor, could reduce the trafficking of lymphocytes in CNS and reduce neuroinflammation. The same rationale is being researched for natalizumab: by selectively inhibiting α4-integrin on the surface of the circulatory lymphocytes, it can prevent lymphocyte aggregation and CNS passage following the onset of ischemia [127,128]. Two clinical trials involving natalizumab administration in IS patients yielded unsatisfactory results [129,130] while multiple clinical trials suggest that fingolimod may be a viable option for reducing post-stroke disability and cerebral infarction volume [131,132,133,134].

Cladribine (CLD) is a purine nucleoside analog that transiently and selectively reduces B and T cells in the periphery. CLD is a prodrug, and it stimulates the death of the said activated cells after phosphorylation, which occurs mainly in the B and T lymphocytes. Since 2017, the EMA approved its use in highly active forms of MS. The main mechanism of action is situated peripherally and consists of the transient suppression of the B cells (in MS, they have a central role in presenting the antigen for T cells and thereafter stimulate the proliferation and reactivation of T cells) and T cells, especially CD4+. Being a very small molecule, CLD crosses the BBB, and it may exert a cytotoxic effect on lymphocytes that have already entered the CNS. As a consequence, it facilitates the durable suppression of the intrathecal humoral response measured by the disappearance of oligoclonal bands [134,135,136].

**Figure 5:**
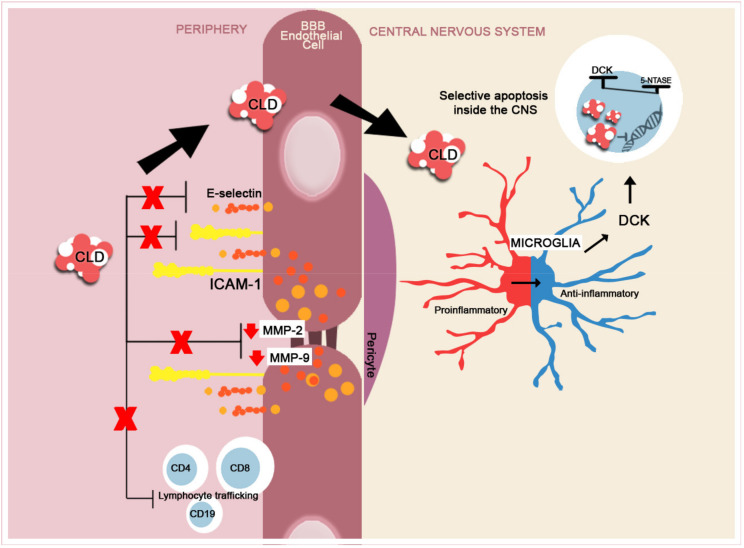
A brief schematic representation of CLD’s mechanism of action. In the peripheral compartment, CLD reduces the expression of adhesion molecules, ICAM-1 and E-selectin and reduces the expression of MMP-2 and -9 [136,137,138,139], thus inhibiting the lymphocyte transition into the CNS. CLD is internalized into the cells and undergoes specific phosphorylation by deoxycytidine kinase (DCK). Immune cells, the lymphocytes being the most susceptible, contain reduced levels of phosphatases 5-nucleotidases (5-NTASE), which will only partially dephosphorylate the accumulated CLD, leading to selected apoptosis and not cellular death (ratio of DCK to 5-NTASE) [140,141]. In MS, CLD selectively reduces Th and B lymphocyte numbers and trafficking from the periphery [142]. CLD reduces the activation of proinflammatory microglia (M1) and shifts the activity towards an anti-inflammatory (M2) phenotype [138].

At the level of the endothelial cells, CLD directly impacts the adhesion molecules that aid in maintaining BBB homeostasis, such as ICAM and E-selectin. It is also supposed to reduce the activity of MMP-2 and 9 and reduce the possibility of lymphocyte transition [137]. CLD also exerts important properties on microglia. Studies on murine models have demonstrated that CLD affects stimulated but not inactive microglia in vitro. It suppresses phagocytosis secondary to microglial activation and appears to modulate gene expression, but not cytokine secretion. This potentially indicates that it aids in shifting from an M1 phenotype, which is highly proinflammatory, to an M2 phenotype, which is anti-inflammatory (Figure 5) [138].

The most widely used agents that address acute inflammation and are extensively used for relapse therapy are glucocorticoids (GC) [143]. The rationale for GC therapy is demonstrated by the important effects they have upon the BBB. They down-regulate the proinflammatory cytokines and inhibit the expression of the adhesion molecules. By directly targeting the Th population of lymphocytes, GC reduce immune cell trafficking through the BBB [144,145]. Studies have demonstrated that administration of methylprednisolone as pulse therapy reduces the secretion of IFNγ, TNFα and IL-2 from peripheral lymphocytes, together with a reduction of CD4+ Th lymphocyte number [146,147]. At the level of the BBB, GC exert down-regulation of the TNFα induced adhesion molecules, VCAM-1 and ICAM-1 [148], E selectin [149], and inhibit MMP-1 and MMP-9 expression [150]. Additionally, GC were proven to induce occludin and claudin expression [150,151,152], restoring the integrity of the BBB. The loss of BBB function can be radiologically assessed by the use of contrast MRI, by determining the T1 contrast enhancing lesions [153].

In Table 1 we summarise the most important effects that certain agents have upon the BBB.

The ideal DMT may traverse the BBB and modify the inflammatory reaction in the periphery and the central compartment. The passage of DMTs through the BBB is not standardly monitored on a non-invasive clinical scale. Measuring the CNS concentration of a DMT is a desideratum that can ameliorate the assessment of personalised treatment. It is not enough to assume that a drug is active in the CNS; we would have to evaluate it. Numerous individual factors underlie the discrepant CSF and plasma concentrations of drugs. One possibility could be fluorinated drugs, with the detection of fluorine signals using MR spectroscopy [154].

The highly selective BBB permeability would purposely impair even the direct in-trathecal administration of immunomodulatory agents due to the uneven distribution and subsequent metabolisation of the subependymal substrate and the parenchyma [155]. The principle of homogenous drug delivery with a good balance between the periphery and the central compartment is currently being considered as an emerging strategy for neurodegenerative and inflammatory CNS pathologies. The advantage of stem cell therapy is shadowed by the limitations of progenitor cells in actively breaking passage through the BBB; nonetheless, the possible mutagenic processes should not be overlooked. Novel gene therapies are based on proposed associations between various viral genes and autologous stem cells, such as lentivirus and adeno-associated virus genes, but the associated risks, such as leukemia and uneven distribution, greatly limit the practical application of these methods [156].

Other therapies with direct effects on and beyond the BBB in MS are under evalu-ation: Bruton’s tyrosine kinase inhibitors, suppressors of the major facilitator super-family domain-containing 2a, MMP inhibitors and long-chain omega-3 acids. There is also an increasing interest in the delivery of active substances in the CNS: the use of nanoparticles, the invasive osmotic opening of the BBB, the use of ‘Trojan horse’ technology (using coupling therapeutic molecules with molecules that use the active transport system through BBB), and the intranasal administration of drugs bypassing BBB. All these initiatives have the main drawback of the quantitatively limited active substance that may be delivered in the CNS, but the presumed desired effects of neuroprotection may be achieved by directly involving the transit function of endothelial cells [9,157,158].

By selectively regulating the endothelial cells to transport specific pharmacological agents, such as avidin-functionalised nanomicelles reactive to cell surface biotin targets, Gonzales-Carter et al. induced an active binding on the surface of selected endothelial cells using anti-PECAM-1 antibodies and the internalisation of the nanomicelles. Therefore, targeting a specific PECAM-1 protein with a high metabolic rate at the level of the BBB cells, the authors demonstrated that the selective impermeability of the endothelial cells can be modulated to allow passage for certain nanoparticles. This innovative technique could contribute to the success of direct and personalised drug administration, maintaining the ideal ratio between the periphery and the CNS concentrations [159].

During the development of DMTs, the passage through BBB was not a priority, and studies have focused on determining whether the body naturally (or pathologically) creates its own transporting ‘vehicles’. Extracellular vesicles play a significant role in transcellular signalling, and they are nothing more than self-secreted nanostructures. Among them, exosomes have been extensively studied because of their ability to carry certain molecules; this places them in the category of highly specific biomarkers and subsequent drug carriers. Their circulatory mechanism and unique properties have been considered as possible targets for glioblastoma diagnosis and treatment [160], but this extends even further into the field of personalised therapy. Studies on murine models showed that by customising the structure of the exosomes, these nanoparticles efficiently carried imposed proteins through the BBB into their targeted cells and the brain parenchyma [161,162]. 

One of the most important unmet needs in MS is developing a DMT to address the progression of the disease, since most agents act upon inflammatory mechanisms. In progressive MS, neuroinflammation has settled in the central compartment and consequently enhances neurodegenerative patterns. In order for a DMT to be efficient in secondary progressive MS, it has to pass the BBB in its active form and directly exert its action upon the CNS inflammatory cells (microglia, astrocyte, oligodendrocyte) [163]. Therefore, in 2019, the FDA approved siponimod for the treatment of secondary-progressive MS. This agent meets the required capabilities of traversing through the BBB by selectively modulating S1P1 receptors (see the drawing above with SPM mechanism of action). Moreover, it doesn’t require additional phosphorylation in order to activate, such as fingolimod [65]. The EXPAND trial researched the effects of siponimod, a more selective S1P modulator and confirmed a 21% reduction in neurological disability in secondary-progressive MS patients compared to placebo [164].

Further extensive transdisciplinary research will unravel the mysteries behind the BBB specificity and hopefully aid in developing the ideal curative molecule and the best way to administer it.

## 7. Conclusions

The BBB is more of a concept than a simple palisade. It is formed by a complex series of epithelial cells with physiological properties that permit an ideal neuronal function. BBB endothelial cells are tightly connected, creating a controlled central environment. The basic components of the BBB, the endothelial cells, are responsible for maintaining a highly specialized homeostasis with the main purpose of securely regulating the external (peripheral compartment) from the internal central compartment. Dysfunction of the BBB is considered an essential step in the initiation and maintenance of the immune attack against the CNS structures and the neurodegenerative process in MS. This dysfunction correlates with the impairment of NVU; the hypoxic hypothesis has been largely studied and accepted lately in the pathologic processes in MS. Hypoxia in MS may be “true” (result from NVU malfunction) or “virtual” (secondary to mitochondrial dysfunction; the inability to properly use oxygen). MS may be a “mitocondriopathy,” as the dysfunction of the mitochondria is found not only in lesions but also in normal-appearing white and gray matter. The disruption of the BBB in MS is heterogenous, transient and reflected during the onset of MS and the aggressiveness of the disease. It is influenced by current therapies and is triggered by less-known factors. Three different but related terms dominate the ongoing neurodegenerative process in progressive forms of MS that are indirectly related to BBB disruption: PIRA, NEDA and smoldering demyelination or silent progression. DMF, modulators of the S1P receptor, cladribine and laquinimod are DMTs that can cross the BBB and exert direct beneficial effects in the CNS through different mechanisms of action. This suggests that combination therapy can be effective in treating MS. The greatest limitation of MS therapy is mirrored in the apparent unflawed and intact BBB, being a hindrance for DMT delivery inside the CNS. Therefore, the research trend is slowly shifting towards the discovery and use of transporter proteins in the form of nanoparticles, gene therapy or the use of the body’s extracellular vesicles. Knowledge about BBB dysfunction may help in the development of effective and stable treatments adequately delivered in the CNS.

## Figures and Tables

**Table 1:** Effects on BBB.

Agent	Effects on the blood–brain barrier
Dimethyl fumarate	↓ expression of adhesion molecules (VCAM-1 ^1^, ICAM-1 ^2^) [84]
Laquinimod	↓ expression of adhesion molecule ICAM-1 [102] ↓ expression of MMP-9 [103]
Fingolimod	↓ S1P1/S1P3 ^4^ expression on the surface of the endothelial cells [110,111] ↓ VEGF ^5^ and ↓ vascular permeability [112]
Cladribine	↓ICAM-1, E-selectin ↓ MMP-2, -9 [136,139]
Natalizumab	Blocks the interaction between the α-4 integrin (lymphocyte surface) and VCAM-1 (endothelial surface) [73]
Interferon-beta	Ensures structural BBB stability in murine models [69,70,71]
Cortisone	↓ expression of adhesion molecules (VCAM-1, ICAM-1, E selectin) [144,148] ↓ MMP-1 and -9 [149] ↑ up-regulation of TJ (occludine, claudin) [150,151]

^1^ Vascular cell adhesion molecule, ^2^ Intracellular Adhesion Molecule, ^3^ Matrix Metalloproteinase, ^4^ Sphingosine-1-Phosphate receptor, ^5^ Vascular Endothelial Growth Factor.
